# Bilateral Central Serous Chorioretinopathy After Treatment of COVID-19 Infection

**DOI:** 10.7759/cureus.23446

**Published:** 2022-03-24

**Authors:** Ali Sharifi, Arash Daneshtalab, Amin Zand

**Affiliations:** 1 Department of Ophthalmology, Shafa Hospital, Kerman University of Medical Sciences, Kerman, IRN

**Keywords:** systemic steroids, central serous chorioretinopathy, ophthalmic manifestations, sars-cov-2 (severe acute respiratory syndrome coronavirus -2), corona virus disease 2019 (covid-19)

## Abstract

We report a rare case of bilateral central serous chorioretinopathy (CSCR) after COVID-19 management with steroids. The patient was a 49-year-old female who presented with bilateral blurred vision three months after the COVID-19 infection. She had been treated with intravenous Remdesivir and Dexamethasone. After her recovery from the disease, she developed gradual visual impairment in both her eyes. Upon examinations and optical coherence tomography, bilateral CSCR was revealed. She was treated with eplerenone (25 mg/day) and propranolol (20 mg/day), and the symptoms were improved after two months. Post-COVID-19 associated CSCR can occur due to steroids administration. Therefore, patients and physicians should be aware of these possible complications and seek an ophthalmology consultation as early as possible.

## Introduction

The current coronavirus disease 2019 (COVID-19) pandemic, which is caused by severe acute respiratory syndrome coronavirus 2 (SARS-CoV-2), can lead to various ocular manifestations. These can be associated with a direct effect of the infection or can be due to the adverse effects of the administered drugs for the treatment of the disease and its sequels. These manifestations include conjunctivitis, episcleritis, uveitis, retinal involvements (chorioretinitis, central serous retinopathy, or retinal vessels occlusions), and papillitis [1,2].

Here, we report a bilateral central serous chorioretinopathy (CSCR) in a patient who had recovered from COVID-19, having been treated with systemic steroids.

## Case presentation

A 49-year-old female presented to our clinic with the blurring of vision in both eyes gradually, about three months after being treated for COVID-19 infection. Her history indicated that she had suffered fever, myalgia, and dyspnea with a positive COVID-PCR test and signs of pneumonitis in her chest computed tomography scan. During her five-day hospital admission, intravenous Remdesivir (200 mg as an initial dose, and then 100 mg daily) and Dexamethasone (4 mg daily) with subcutaneous low-molecular-weight heparin (50 mg twice daily) for prophylaxis of thrombosis had been administrated, and she had been discharged with prednisolone 10 mg twice daily for two weeks.

**Figure 1:**
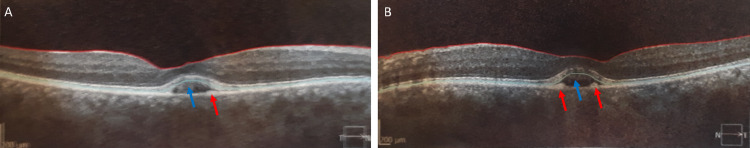
Optical coherence tomography (OCT) of right (A) and left (B) eyes showed serous macular detachment, with an accumulation of subretinal fluids (SRF, blue arrows) and retinal pigment epithelium detachment (PED, red arrows).

Office examination revealed a best-corrected visual acuity (BCVA) of 20/40 in the right eye and 20/50 in the left eye. Pupillary reactions were normal, with a negative relative afferent pupillary defect (RAPD). Intraocular pressures (IOP) were within the normal range. In slit-lamp examinations, the anterior segment examinations were unremarkable without any signs of inflammation. On dilated fundus exam, vitreous media was clear in both eyes. Bilateral serous detachment of the macula was revealed. In optical coherence tomography (OCT), serous detachment of the macula, accumulation of subretinal fluid (SRF), and retinal pigment epithelial detachment (PED) were demonstrated in both eyes (Figure 1A, 1B).

The patient was diagnosed with bilateral CSCR and was treated with propranolol 20 mg and eplerenone 25 mg daily for one month. The symptoms were resolved about two months later, and the BCVA of both eyes improved to 20/25.

## Discussion

Previous studies showed SARS-CoV-2 might bind to the angiotensin-converting enzyme (ACE) 2 receptor of human cells. ACE 2 receptors are present in all major organs, especially the lungs, heart, and endothelium of vessels. Therefore, SARS-CoV-2 can cause endotheliitis leading to endothelial dysfunction [3]. The exact pathophysiology of CSCR remains unknown. Hypothetically, the disease can occur due to endothelial dysfunction and hyperpermeability of Choriocapillaris, and also retinal pigment epithelium (RPE) pump dysfunction [4]. However, there is no evidence that SARS-CoV-2 can lead to CSCR directly.

CSCR is a known complication of steroid administration, irrespective of the drug dose or its administration route. It can occur for a few days to months after the drug initiation [4]. Steroids (intravenously, orally, or an inhaler) are commonly used in the management of patients with COVID-19, and all previous reports of post-COVID-19 associated CSCR had a history of taking steroids [2,5-7]. Therefore, the most probable etiology of the post-COVID-19 associated CSCR is maybe steroid use. Most of these reports described unilateral CSCR with spontaneous improvement and without any interventions [2,5,6]. Our patient had bilateral CSCR after COVID-19 management with steroids. The bilateral presentation of the disease has not been much reported yet [7,8]. Weenink et al. [9] showed in 52% of patients with bilateral CSCR, at least one relative was affected and suggested a genetic predisposition to CSCR. But our patient had a negative family history for a similar scenario. Bilateral CSCR is more common in patients over 50 years of age (our patient's age was 49 years) [10].

Acute CSCR is a self-limited condition with spontaneous reattachment of neurosensory retina and absorption of SRF during 2-3 months in the majority of cases. In about 15% of patients, SRF may remain for more than six months, which is described as chronic CSCR. Patients with chronic or recurrent CSCR need interventions, including administration of anti-steroid (e.g., eplerenone, or spironolactone), anti-adrenergic (e.g., propranolol, or metoprolol), or photodynamic therapy [11]. We decided to treat the patient medically with eplerenone and propranolol due to the bilateral involvement of the disease. She had an improvement in her visual symptoms after about two months.

## Conclusions

Patients with a history of COVID-19 management and their physicians should be aware of possible ophthalmic complications of the administrated drugs, especially steroids, and seek an ophthalmology consultation on time. In addition, introducing protocols for controlling the load of steroid administration in COVID-19 patients is necessary to reduce these complications.
